# Response to letter to the editor: Awareness strategies and the physiological impact of pain in prehospital analgesia management

**DOI:** 10.1186/s13049-025-01353-y

**Published:** 2025-07-01

**Authors:** Pär Wennberg, Glenn Larsson, Kristoffer Wibring

**Affiliations:** 1 PreHospen – Centre for Prehospital Research, University of Borås, Borås, Sweden; 2 Research, Education, Development and Innovation Department, Skaraborg Hospital, Skövde, Sweden; 3 School of Health Sciences, Jönköping University, Jönköping, Sweden; 4 School of Health Sciences, University of Skövde, Skövde, Sweden; 5 PICTA, Prehospital Innovation Arena, Lindholmen Science Park, Gothenburg, Sweden; 6 Department of Ambulance and Prehospital Care, Region Halland, Halmstad, Sweden; 7 Institute of Health and Care Sciences, Sahlgrenska Academy, University of Gothenburg, Gothenburg, Sweden

We would like to thank the authors of their letter to the editor “*Awareness Strategies and the Physiological Impact of Pain in Prehospital Analgesia Management*” for their thoughtful and insightful comments on our study. We appreciate their recognition of the significance of our findings and their emphasis on improving pain management in prehospital care. The challenges they highlight align with the broader discussions on pain assessment and analgesia management, and we acknowledge the importance of the four key issues outlined by the Royal College of Emergency Medicine.

Our study reinforces the notion that currently validated pain assessment tools often have limited applicability in acute and prehospital settings [1]. As the authors of the letter to the editor correctly point out, there is a need for patient-centered, reliable medications and application methods, supported by objective criteria. One potential solution to this challenge is the increased use of behavioral pain assessment tools. While not a complete solution, such tools have shown promise in addressing pain assessment difficulties, particularly in cases where self-reporting is problematic. Previous studies indicate that the Behavioral Rating Scale (BRS), despite not being fully validated, has been used in clinical ambulance work [2–5]. In one of these studies, ambulance nurses could choose between the Numerical Rating Scale (NRS) and BRS for pain assessment [4]. Notably, 64% of the 1,426 patients were assessed using BRS, despite NRS being the recommended default tool. This calls for the development of validated pain assessment tools as alternatives that may better fit the complexities of prehospital and emergency care.

We also strongly support the authors’ emphasis on integrating pain assessment into documentation systems used in ambulances. Incorporating pain scores systematically alongside demographic data and vital signs would enhance the continuity and consistency of pain management. Additionally, we agree with the need for further investigation into the relationship between pain assessment and vital parameters in Early Warning Scores (EWS). Given the link between pain and physiological responses, we argue that pain assessment should be more explicitly integrated into EWS. Recognizing pain as a fundamental component of EWS could strengthen its role in clinical decision-making and improve patient outcomes by ensuring that pain management receives the attention it deserves.

We sincerely appreciate the discussion brought forth by the authors and the opportunity to engage in this critical conversation. We hope that ongoing research will continue to refine and optimize prehospital pain assessment and management strategies.

Sincerely,

Pär Wennberg, Glenn Larsson & Kristoffer Wibring.

## Data Availability

No datasets were generated or analysed during the current study.
